# A transient reporter for editing enrichment (TREE) in human cells

**DOI:** 10.1093/nar/gkz713

**Published:** 2019-08-20

**Authors:** Kylie Standage-Beier, Stefan J Tekel, Nicholas Brookhouser, Grace Schwarz, Toan Nguyen, Xiao Wang, David A Brafman

**Affiliations:** 1 School of Biological and Health Systems Engineering, Arizona State University, Tempe, AZ 85287, USA; 2 Molecular and Cellular Biology graduate program, Arizona State University, Tempe, AZ 85287, USA; 3 Graduate Program in Clinical Translational Sciences, University of Arizona College of Medicine-Phoenix, Phoenix, AZ 85004, USA

## Abstract

Current approaches to identify cell populations that have been modified with deaminase base editing technologies are inefficient and rely on downstream sequencing techniques. In this study, we utilized a blue fluorescent protein (BFP) that converts to green fluorescent protein (GFP) upon a C-to-T substitution as an assay to report directly on base editing activity within a cell. Using this assay, we optimize various base editing transfection parameters and delivery strategies. Moreover, we utilize this assay in conjunction with flow cytometry to develop a transient reporter for editing enrichment (TREE) to efficiently purify base-edited cell populations. Compared to conventional cell enrichment strategies that employ reporters of transfection (RoT), TREE significantly improved the editing efficiency at multiple independent loci, with efficiencies approaching 80%. We also employed the BFP-to-GFP conversion assay to optimize base editor vector design in human pluripotent stem cells (hPSCs), a cell type that is resistant to genome editing and in which modification via base editors has not been previously reported. At last, using these optimized vectors in the context of TREE allowed for the highly efficient editing of hPSCs. We envision TREE as a readily adoptable method to facilitate base editing applications in synthetic biology, disease modeling, and regenerative medicine.

## INTRODUCTION

The rapid advancement of CRISPR/Cas-based technologies has allowed for the modification (i.e. deletion, mutation and insertion) of human cells at precise genomic locations (1–3). For applications in which precise editing of a single nucleotide is desired, the CRISPR/Cas machinery can be used to introduce site-specific double-stranded breaks (DSB) followed by homology-directed repair (HDR) using an exogenous DNA template (4). However, HDR is inefficient in mammalian cells, especially in recalcitrant cells such as human pluripotent stem cells (hPSCs), and repair of DSB is predominantly achieved through non-homologous end joining (NHEJ) (5–9). In addition, NHEJ results in insertion or deletion of nucleotides (indels), resulting in undesired disruption (e.g. frameshift mutations, premature stop codons, deletion) of the targeted genes.

As an alternative to standard gene editing approaches that require a DSB, several groups have reported the development of deaminase base editors that do not rely on HDR to introduce single nucleotide genomic changes (10). Broadly speaking, these base editors consist of a fusion of three components—a D10A nickase Cas endonuclease, cytidine deaminase (APOBEC1), and a DNA uracil glycosylase inhibitor (UGI). This complex is capable of converting cytosine to thymine (11) (or adenine to guanine on the complementary strand) (12) without the need for a DSB and homology repair template. More specifically, after sgRNA-mediated targeting of the Cas9^D10A^ nickase to the desired loci, APOBEC1 catalyzes the deamination of cytidine to uracil. During replication, DNA polymerase will incorporate thymidine at this position because it has the same base paring properties as uracil. Typically, the base excision repair pathway through the activation of uracil DNA glycosylase would remove the uracil and replace it with a cytidine. As such, the UGI prevents such reversion to a cytidine from occurring. At last, the nicking of the non-edited strand through the action of the Cas9^D10A^ nickase will stimulate DNA repair using the edited strand as the template. Overall, genome modification through the use of base editors has been shown to result in formation of fewer indels when compared to HDR-based methods (13,14).

Despite the advantages that deaminase base editors offer, identification and isolation of cell populations that have been successfully edited remains challenging. Specifically, there is no readily detectable phenotype to distinguish edited from unedited cells. In turn, isolation of edited cell populations requires single cell isolation followed by downstream sequencing verification (15). Some progress has been made to help enrich for edited cells, such as co-transfecting plasmids with a fluorescent reporter and using flow cytometry to isolate reporter-positive cells. Similarly, fluorescent protein conversions have been used to report on gene editing activity and enrich for cell populations with single base edits (16,58).

In this work, we sought to develop an assay to allow for the real-time, fluorescent-based identification and isolation of base-edited cell populations. To develop this method, we were motivated by previous work that employed a genomically integrated green fluorescent protein (GFP) that is converted to blue fluorescent protein (BFP) upon CRISPR/Cas9-driven HDR (16). Here, we engineered a BFP variant that undergoes conversion to GFP after targeted modification with a cytidine deaminase-based DNA base editor. We applied our BFP-to-GFP conversion assay to optimize various base editing transfection parameters and delivery strategies. We then utilized this BFP-to-GFP assay in conjunction with flow cytometry to develop a technique called transient reporter for editing enrichment (TREE) which allows for the fluorescent-based isolation of base edited cell populations. As such, we applied TREE to enrich for cell populations that had been edited at various genomic loci, including sites that are refractory to modification. Significantly, we demonstrate how TREE enables enrichment of edited human pluripotent stem cells (hPSCs), a cell type that is resistant to traditional CRISPR/Cas9 HDR-based approaches and in which modification via base editors has not been previously reported. Overall, because TREE can be facilely implemented to isolate edited cell populations, it will significantly enhance and enable the use of base editors for numerous downstream applications including those in synthetic biology, protein engineering, disease modeling and regenerative medicine.

## MATERIALS AND METHODS

### Plasmid construction

Unless otherwise noted, all molecular cloning polymerase chain reactions (PCR) were performed using Phusion^®^ High-Fidelity DNA polymerase (New England Biolabs, Ipswich, MA, USA) using the using the manufacturer's recommended protocols. All restriction enzyme (New England Biolabs) digests were performed according to the manufacturer's instructions. Ligation reactions were performed with T4 DNA Ligase (New England Biolabs) according to the manufacturer's instructions. PCR primers and oligonucleotides were synthesized by Integrated DNA Technologies (Coralville, IA, USA). All PCR products and intermediate plasmid products were confirmed via Sanger sequencing (DNASU Sequencing Core Facility and Genewiz). Complete plasmid sequences will be made available upon request.

For construction of the pEF-BFP plasmid, we utilized PCR to add the H-66 and protospacer adjacent motif (PAM) site mutations into a GFP cassette (Addgene #11154). PCR products containing these mutations were digested with SapI/EcoRI and SapI/NotI and ligated into a EcoRI/NotI digested EF1α expression vector (Addgene #11154).

For construction of the pDT-sgRNA vector, sgRNAs were synthesized as pairs of oligonucleotides (Supplementary Table S1). Subsequently, 5′ phosphates were added to each oligonucleotide pair by incubating 1 μg oligonucleotide in 50 μl reactions containing 1× T4 DNA Ligase Buffer (New England Biolabs) and 10 units of T4 Polynucleotide Kinase at 37°C overnight. Oligonucleotides were then duplexed by heating the kinase reactions to 90°C on an aluminum heating block for 5 min followed by slowly returning the reaction to room temperature over 1 h. Following duplexing, guides were cloned into a modified pSB1C3 vector containing a U6 promoter, inverted BbsI restriction enzyme digestion sites, and a *Streptococcus pyogenes* recognized sgRNA hairpin. For construction of pMT-sgRNA, pairs of sgRNAs (Supplementary Table S1) were PCR amplified with primers adding EcoRI/SapI restriction enzyme digestion sites or SapI/XbaI restriction enzyme digestion sites. Purified PCR products were then digested with the respective restriction enzymes and ligated into EcoRI/XbaI digested pUC19 vector (Addgene #50005). The resultant vector contained pairs of sgRNA expression cassettes. To add additional sgRNA expression cassettes, pairs of sgRNAs were PCR amplified with primers that add HindIII/SapI or SapI/HindIII restriction enzyme digestion sites. These products were then digested with HindIII/SapI and ligated into HindIII digested and dephosphorylated pDT-sgRNA vector.

For insertion of the EF1α promoter into the pCMV-BE4-Gam (Addgene #100806) and pCMV-AncBE4max (Addgene #112094), EF1α was PCR amplified from an EF1α expression vector (Addgene #11154) adding SpeI/NotI restriction enzyme digestion sites. After purification and digestion, these PCR products were ligated into SpeI/NotI digested and dephosphorylated pCMV-BE4-Gam or pCMV-AncBE4max vectors.

### Cell culture

All media component were purchased from ThermoFisher Scientific (Waltham, MA, USA) unless indicated otherwise. HEK293 cells were cultured on poly-L-ornithine (4 μg/ml; Sigma Aldrich, St Louis MO, USA) coated plates in the following media: 1× high glucose Dulbecco's modified Eagle's medium, 10% (v/v) fetal bovine serum, 1% (v/v) L-glutamine penicillin/streptomycin. Culture medium changed was every other day and cells were passaged with Accutase (ThermoFisher) every 5 days. HPSCs were cultured on 12-well tissue culture plates coated with Matrigel™ (BD Biosciences, San Jose, CA, USA) in Essential 8™ Medium (E8) (ThermoFisher). HPSCs were cultured in mTESR1 medium (STEM CELL Technologies). Culture medium was changed everyday and cells were passaged with Accutase every 4–5 days. After passaging, the medium was supplemented with 5 μM Rho kinase inhibitor (ROCKi; Y-27632 [BioGems, Westlake Village, CA, USA]) for 24 h to aid in single cell survival.

### Isolation of episomal DNA

After 48 h following transfection, HEK293 cells were dissociated from the tissue plates with Accutase, washed twice with phosphate-buffered saline and resuspended in RNAse-A containing solution. Cells were then lysed via alkaline lysis and the resultant debris was precipitated via centrifugation at 1.2 × 10^4^ × *g* for 10 min. Supernatant DNA was isolated by column DNA purification using the manufacture recommended protocol (Sigma-Aldrich: NA0160).

### Generation of HEK293-BFP line

The HEK293T-BFP cell line was generated via homology independent targeted integration (HITI) (17). Briefly, the BFP coding sequence was PCR amplified with primers adding EcoRI restriction enzyme digestion sites. The resultant PCR product was EcoRI digested, phosphorylated and ligated into an EcoRI/SmaI digested vector containing an EF1α promoter, puromycin resistance cassette and HITI protospacer sequence (pEF-BFP-Puro^R^). The pEF-BFP-Puro^R^ vector was co-transfected in HEK293s with pX330 (Addgene #42230) and a custom sgRNA vector (pHSG(*C1ORF228*)-1C3) targeting the *C1ORF228* locus. Transfections were conducted in a 24-well plate with 300 ng pX330, 400 ng pEF-BFP-Puro^R^, 50 ng sgRNA vector, 1.5 μl Lipofectamine 3000 (ThermoFisher Scientific) and 1 μl P3000 transfection reagent. Cells were passaged at 72 h post-transfection into a single well of a 6-well plate and selected with 0.5 μg/ml puromycin for 2 weeks.

### RNP complex formation

For purification of recombinant BE3 (rBE3) protein, BL21 Star DE3 cells (ThermoFisher) were transformed with pET42b-BE3 (Addgene #87437). Protein expression was induced for 18 h in 2L baffled flasks at 16°C with 0.5 mM isopropyl β-d-1-thiogalactopyranoside (IPTG). Cells were then harvested by centrifugation followed by lysis by sonication in lysis buffer [50 mM NaH_2_PO_4_ (pH 8.0), 300 mM NaCl, 10 mM imidazole, 1% Triton X-100, 1 mM Dithiothreitol (DTT) and 1 mg/ml lysozyme]. The lysate was cleared by centrifugation at 10 000 *g* for 30 min at 4°C. The supernatant was incubated with 2 ml Ni-NTA beads (Qiagen, Germantown, MD, USA) equilibrated in lysis buffer for 1 h at 4°C, followed by washing with 5 ml wash buffer [50 mM NaH_2_PO_4_ (pH 8.0), 300 mM NaCl and 20 mM imidazole] three times. BE3 protein was eluted with 1 ml elution buffer [50 mM Tris–HCl (pH 7.6), 250 mM NaCl and 0.2 M imidazole]. The purified BE3 protein was exchanged and concentrated with storage buffer [20 mM HEPES (pH 7.5), 150 mM KCl, 1 mM DTT and 10% glycerol] using an Ultracel 100K cellulose column (Millipore, Burlington MA, USA). The concentration of the protein was determined by sodium dodecyl sulphate-polyacrylamide gel electrophoresis using bovine serum albumin standards.

Synthetic sgRNAs were synthesized as 2′-O-methylated sgRNAs (Synthego, Menlo Park, CA, USA). sgRNA was resuspended in ddH_2_0 to a concentration of 100 μM. Concentrated rBE3 (∼1 μM) was supplemented with 10 mM MgCl_2_, followed by addition of a 3:1 molar ratio of sgRNA. The solution was incubated at room temperature for 15 min to allow BE3–sgRNA complex formation.

### Cell transfections

For plasmid-based transfections HEK293 cells were transfected in 12-well tissue culture plates at 40% confluence with the following reagents per well: 600 ng pCMV-BE4-Gam, 200 ng pEF1α-BFP and 200 ng sgRNA vector [sg(BG), sg(NT), pDT-sgRNA or pMT-sgRNA], 1.5 μl Lipofectamine 3000 Transfection Reagent (ThermoFisher) and 2 μl P3000 reagent (Thermo Fisher). For RNP-based transfections, complexed BE3-RNPs were incubated with transfection reagents for 10–15 min and added dropwise to each well at a final concentration 250 nM in 250 μl volume total. HPSCs were transfected on 12-well tissue culture plates with 900 ng of base editing vector (pCMV-BE4-Gam, pCMV-AncBE4max, pEF1α-BE4-Gam or pEF1α-AncBE4max), 300 ng, pEF1α-BFP, 300 ng pDT-sgRNA and 4 μl Lipofectamine Stem Transfection Reagent (ThermoFisher). All cells were harvested for sorting and/or analysis 48 h post-transfection

### Fluorescence microscopy

All imaging was performed on a Nikon Ti-Eclipse inverted microscope with an LED-based Lumencor SOLA SE Light Engine using a Semrock band pass filter. GFP was visualized with an excitation at 472 nm and emission at 520 nm. BFP was visualized with the DAPI fluorescence channel with excitation at 395 nm and emission at 460 nm.

### Flow cytometry

Cells were dissociated with Accutase for 10 min at 37°C, and passed through a 40 μm cell strainer. Cells were then washed twice with flow cytometry buffer (BD Biosciences) and resuspended at a maximum concentration of 5 × 10^6^ cells per 100 μl. Flow cytometry analysis was performed on an ACCURI C6 (BD Biosciences). Flow cytometry sorting was performed on a FACSAria IIu. Flow cytometry files were analyzed using with FACSDiva software (BD Biosciences), FlowJo (FlowJo LLC, Ashland, OR, USA), and custom Matlab (MathWorks, Natick, MA, USA) script.

### Quantification of base editing efficiency

For HEK293 cells, genomic DNA (gDNA) was extracted from sorted and unsorted cells using NucleoSpin kit (Macherey Nagel, Bethlehem, PA, USA). PCR was performed with 500 ng gDNA in a 50 μl reaction with Phusion^®^ High Fidelity DNA polymerase (New England Biolabs) using the primers listed in Supplementary Table S2 and PCR protocols listed in Supplementary Table S3. HPSCs were directly sorted into a 50 μl master mix consisting of 1× Phire Hot Start II DNA Polymerase (ThermoFisher), 1 μM forward primer and 1 μM reverse primer. PCR was performed using the following conditions: 98°C for 5 min, followed by 40 cycles at 98°C for 5 s, 56°C for 5 s and 72°C for 20 s, followed by a final 5 min 72°C extension. All products sizes were confirmed on an agarose gel prior to Sanger sequencing. Sanger sequencing was performed using column purified PCR products with primers listed in Supplementary Table S2. Base editing efficiencies were analyzed from Sanger sequence chromatograms using EditR (18) using the parameters listed in Supplementary Table S4.

### Off-target analysis

For the data presented in Figure 4, analysis was performed for the top off-target loci for sgRNAs for genomic sites 1–3 as predicted using GUIDE-seq (19). sg(BG) genomic off-targets were predicted *in silico* via CCTop using default parameters for *S. pyogenes* Cas9 against human genome reference sequence hg38 (20). Quantification of base editing efficiency at these off-target sites was performed in a similar manner to that at on-target sites. The PCR primers used to analyze these off-target sites are presented in Supplementary Table S5.

### Clonal isolation of edited HEK293 cells

HEK293 cells were transfected in 12-well tissue culture plates at 40% confluence with the following reagents per well: 600 ng pCMV-BE4-Gam, 200 ng pEF1α-BFP, 200 ng pMT-sgRNA, 1.5 μl Lipofectamine 3000 Transfection Reagent and 2 μl P3000 reagent. After 48 h, cells were dissociated with Accutase for 5 min at 37°C, triturated and passed through a 40 μm cell strainer. Cells were then washed twice with flow cytometry buffer (BD Biosciences) and resuspended at a maximum concentration of 5 × 10^6^ cells per 100 μl. Single GFP+ cells were sorted into a single well of a 96-well plate and expanded to a 24-well plate prior to analysis.

### Next-generation sequencing (NGS) of PCR amplicons

After gDNA isolation, PCR was performed using the NGS primers listed in Supplementary Table S6 and PCR protocols listed in Supplementary Table S7. PCR amplification was carried out using Phusion^®^ High Fidelity DNA polymerase (New England Biolabs) as described above. The products were column purified using the QIAquick PCR purification kit (Qiagen). Samples were sequenced on an Illumina MiSeq by GENWIZ. Reads were trimmed for high quality sequences via BBDuk adapter/quality filtering tool of the BBtools suite. Reads below a threshold quality score of 31 were removed using the following command (bbduk.sh in = ‘$i’ out = ‘$x’_trim.fastq.gz trimq = 30 minlen = 250), where ‘i’ is the sample file and ‘x’ is the base name of the respective input sample file. Trimmed FASTQ files were analyzed for C-to-T editing outcomes via custom python script (Python Software Foundation).

### Statistical analysis

Unless otherwise noted, all data are displayed as mean ± standard deviation (S.D). Pairwise comparisons were made using Student's *t*-test and multiple comparisons were made using ANOVA statistical methods.

## RESULTS

### BFP-to-GFP conversion allows for detection of base-editing activity

**Figure 1.**
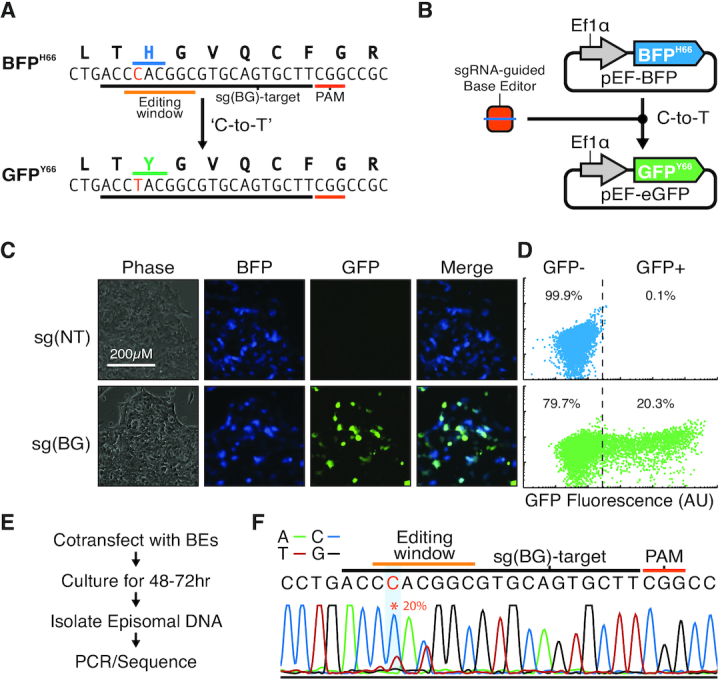
Conversion of BFP to GFP enables detection of base-editing activity in cells. (**A**) A mutant BFP was designed to convert to GFP upon a C-to-T nucleotide conversion. The protospacer sequence (underlined black) for the sgRNA, sg (BG), targeting the ‘CAC’ codon (underlined blue) resulting in a C-to-T conversion to ‘TAC’ (underlined green) and the corresponding amino acid change of histidine (blue) to tyrosine (green) at the 66th amino acid position in BFP. A PAM (underlined red) was placed in the position to orient the base editing window (underlined orange) around the C nucleotide (red) to facilitate BFP^H66^ to GFP^Y66^ conversion. All alternative C-to-T conversions in the editing window resulted in silent mutations of the coding sequence. (**B**) The BFP mutant was cloned into a vector, pEF-BFP, with a human EF1α promoter driving expression. Targeting pEF-BFP with a cytidine deaminase base editor results in a C-to-T conversion causing a shift in the fluorescent emission spectra from BFP to GFP. (**C**) Representative fluorescent microscopy images of HEK293 cells transfected with pEF-BFP, pCMV-BE4-Gam and sg(NT) (top row) or sg(BG) (bottom row). (**D**) Representative flow cytometry plots of HEK293 cells transfected with pEF-BFP, pCMV-BE4-Gam and sg(NT) (top) or sg(BG) (bottom). *Y*-axis is a non-fluorescent control channel. (**E**) Schematic for isolation and detection of editing of episomal DNA after transfection. (**F**) Representative Sanger sequencing chromatogram of amplicons of episomal DNA isolated from HEK293 cells transfected with pEF-BFP, pCMV-BE4-Gam and sg(BG). The presence of T-nucleotide (red trace) at the target nucleotide (red asterisk) demonstrates the C-to-T base conversion responsible for the amino acid change of histidine to tyrosine at the 66th amino acid position and subsequent shift of the BFP emission spectra of the resultant protein to a GFP variant.

To establish that BFP to GFP conversion could be used as the basis for an assay to detect genomic base editing, we utilized a BFP mutant that converts to a GFP upon a C-to-T nucleotide conversion (Figure 1A). Briefly, this BFP mutant (BFP^H66^) contains a histidine at the 66th amino acid position encoded by a ‘CAC’ codon. The C-to-T conversion of that codon to ‘TAC’ or ‘TAT’ will result in an amino acid change from a histidine to a tyrosine. In turn, this amino acid change will cause a shift of the emission spectra of the resultant protein generating a GFP variant (GFP^Y66^) (21). Because the optimal nucleotide base editing window is typically 12–18 nt upstream from the PAM, we also placed a *S. pyogenes* Cas9 PAM ‘NGG’ in a position that would enable base editing to occur at the target ‘CAC’ codon. To verify the utility of this fluorescent protein to report on base editing activity, we cloned the BFP coding sequence into a vector with a human EF1α promoter to drive expression (pEF-BFP; Figure 1B). In addition, we designed a sgRNA vector [sg(BG)] that would direct the base editing machinery to the target ‘CAC’ codon resulting in a C-to-T conversion and the subsequent amino acid change of histidine to tyrosine at the 66th amino acid position (Figure 1A). HEK293 cells were co-transfected with pEF-BFP, a base editing vector (pCMV-BE4-Gam) and sg(BG) or a control non-targeting sgRNA [sg(NT)]. Fluorescent microscopy (Figure 1C) and flow cytometry (Figure 1D) revealed that targeting pEF-BFP with sg(BG) resulted in the generation of BFP/GFP double positive cells. However, targeting pEF-BFP with sg(NT) did not result in the generation of any BFP/GFP positive cells. To confirm GFP expression was a consequence of direct editing of the target codon in pEF-BFP, we implemented a strategy to isolate and detect editing of episomal DNA after transfection (Figure 1E). Sanger sequencing of isolated pEF-BFP DNA established that editing had occurred at the target ‘CAC’ codon in pEF-BFP resulting in a change to ‘TAC’ or ‘TAT’ reflected in the GFP emission (Figure 1F). Overall, these results confirm that the GFP-to-BFP conversion corresponds to C-to-T conversion at targeted base editing sites.

**Figure 2.**
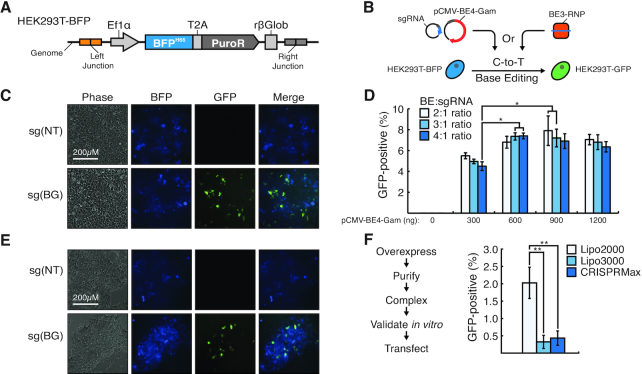
BFP-to-GFP conversion reports on base-editing at a chromosomal locus. (**A**) A pEF-BFP-Puro^R^ vector was integrated into the *C1ORF228* locus using homology-independent targeted integration to generate the HEK293-BFP cell line. (**B**) Schematic for plasmid or RNP base editing optimization using the HEK293-BFP line. (**C**) Representative fluorescent microscopy images of HEK293-BFP cells transfected with 600 ng pCMV-BE4-Gam and 200 ng sg(NT) (top row) or sg(BG) (bottom row). Scale bar = 200 μm. (**D**) Editing efficiencies (GFP-positive cells) of HEK293-BFP cells transfected with various amounts of pCMV-BE4-Gam and ratios with the sg(BG) vector. *n* = 3, * = *P* < 0.05. (**E**) Representative fluorescent microscopy images of HEK293-BFP cells transfected with BE3-sg(BG) or –sg(NT) RNP complexes. (**F**) Schematic for RNP complex generation and transfection. BE3 was overexpressed, purified, complexed and validated *in vitro*, and transfected. Editing efficiencies (GFP-positive cells) of HEK293-BFP cells transfected with RNP complexes using various delivery reagents. *n* = 3, * = *P* < 0.05, ** = P < 0.01.

Next, we wanted to establish that the BFP-to-GFP conversion would correlate with base-editing efficiency at a chromosomal locus. To that end, we employed a HEK-293 cell line (herein referred to as HEK293-BFP) in which BFP^H66^ was stably integrated into a known genomic location (C1ORF228; Figure 2A). We then used this line to enable the analysis of the efficiency of base editing genomic loci (Figure 2B). To first assess plasmid-based base editing, we co-transfected pCMV-BE4-Gam and sg(BG) plasmid DNA in HEK293-BFP cells. Targeting with sg(BG), but not sg(NT), resulted in generation of detectable GFP+ cells, indicating successful base editing at the targeted genomic loci (Figure 2C). Moreover, we were able to use this assay to systematically evaluate genomic base editing efficiencies using a range of pCMV-BE4-Gam plasmid amounts at varying ratios with the sg(BG) vector (Figure 2D). This analysis revealed that base editing plasmid concentration and base editor to sgRNA ratios could enhance genomic base editing efficiencies approximately 2-fold. Because ribonucleoprotein (RNP) complex-based strategies have been previously shown as an attractive alternative to plasmid-based Cas9 genome engineering (22–24), we also utilized BFP-to-GFP conversion as an assay to optimize RNP-driven base editing. As such, we generated RNPs through the *in vitro* complexing of purified base editing protein with sg(BG) or sg(NT) (Figure 2E). Our initial analysis revealed that RNP delivery using the same transfection reagent that was used for plasmid delivery of the base editor (i.e. Lipofectamine™ 3000) did not result in substantial BFP-to-GFP conversion (Figure 2F). In turn, we utilized BFP-to-GFP to evaluate various commercially available transfection reagents to optimize RNP delivery for base editing applications. From this analysis, we were able to determine that Lipofectamine™ 2000 allowed for a >4-fold increase in genomic base editing efficiency compared to other commercially available reagents such as Lipofectamine™ and CRISPRMAX (Figure 2F). Despite this, RNP-driven delivery was about 4-fold less efficient in genomic base editing compared to plasmid delivery. Thus, for the remainder of this study we proceeded with plasmid delivery of base editors. Nonetheless, this collective data demonstrates that BFP-to-GFP conversion correlates to base editing efficiency at genomic loci. Moreover, this approach allows for the facile and systematic optimization of base editing in human cells using plasmid- and RNP-based approaches.

### Development of transient reporter for editing enrichment (TREE) to identify and efficiently isolate base-edited cell populations

**Figure 3.**
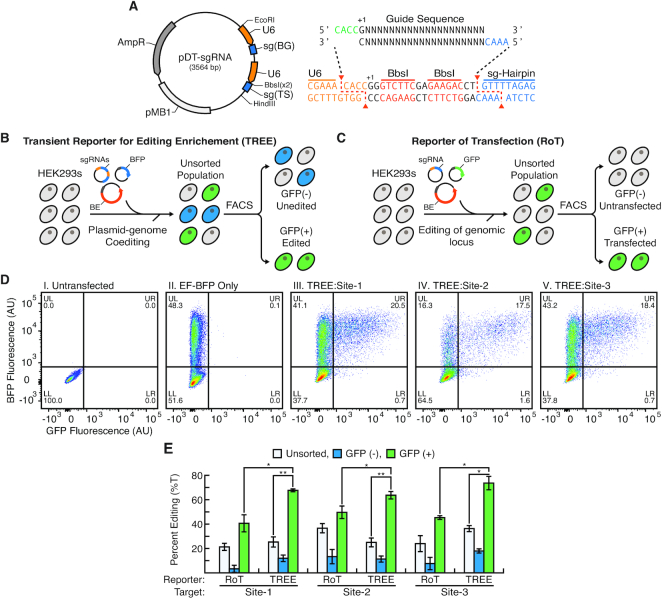
Enrichment of base-edited cell populations using TREE. (**A**) Plasmid map of pDT-sgRNA vector that contains sg(BG) and sg(TS). Expression for both sgRNA cassettes is driven by separate U6 promoters (orange arrows). The BbsI restriction sites allow for direct restriction enzyme-based cloning of new target sites. (**B**) Schematic for enrichment of edited cells using TREE. HEK293 cells are co-transfected with pEF-BFP, pCMV-BE4-Gam and pDT-sgRNA vectors. After 48 h post-transfection, flow cytometry is used to sort cell populations into GFP-positive and -negative fractions. (**C**) Schematic for enrichment of edited cells using reporter of transfection (RoT). HEK293 cells are co-transfected with pEF-GFP, pCMV-BE4-Gam and sg(TS) vectors. After 48 h post-transfection, flow cytometry is used to sort cell populations into GFP-positive and -negative fractions. (**D**) Representative flow cytometry plots of (i) untransfected HEK293 cells and (ii) HEK293 cells transfected with pEF-BFP only as well as HEK293 cells in which TREE was applied targeting (iii) Site-1, (iv) Site-2 and (v) Site-3. (**E**) Quantification of base editing efficiency at Site-1, Site-2 and Site-3 in GFP-positive, GFP-negative and unsorted cell populations isolated using TREE- or RoT-based enrichment strategies. *n* = 3; * = *P* < 0.05, ** = *P* < 0.01.

Conventional base editing approaches that use reporters of transfection (herein abbreviated as RoT) only report on the efficiency of plasmid delivery to a cell but not directly on the efficiency of base editing within these cells. As such, we hypothesized that we could employ BFP-to-GFP conversion, which directly correlates to base editing activity within a cell, as a TREE to allow for the identification and enrichment of cells in which targeted genomic base editing had occurred. To facilitate this, we engineered a dual-targeting sgRNA (pDT-sgRNA) vector that contains both sg(BG) and a sgRNA for a genomic target site [sg(TS)] (Figure 3A). Moreover, the pDT-sgRNA vector was designed to allow for the facile cloning of new target sites via BbsI restriction enzyme digestion and ligation of sg(TS) oligonucleotides (Figure 3A). Accordingly, we designed pDT-sgRNA vectors with sequences targeting three genomic locations (Sites 1–3). To utilize TREE for enrichment of cells that have been edited at specific loci, we co-transfected these pDT-sgRNA vectors with pEF-BFP and pCMV-BE4-Gam into HEK293 cells using the optimized base editing parameters identified using the BFP-to-GFP conversion assay (Figure 3B). Flow cytometry was then used to isolate GFP-positive and -negative cells. For comparison, we used a conventional RoT as a strategy to enrich for edited cell populations (Figure 3C). Specifically, after co-transfecting HEK293 cells with pEF-GFP and sg(TS) plasmids, we used flow cytometry to sort for GFP-positive and -negative cell populations. Flow cytometry analysis of cells in which TREE was applied confirmed the presence of BFP and GFP-positive cell populations indicative of active base editing (Figure 3D). Importantly, in these cell populations there was also a significant percentage of cells that were BFP-positive but GFP-negative, suggesting that isolating cell populations exclusively based upon a reporter of transfection would significantly limit the enrichment of edited cells. To confirm this, we performed Sanger sequencing of the targeted genomic sites in GFP-positive, GFP-negative and unsorted cell populations isolated from TREE and RoT approaches (Figure 3E and Supplementary Figure S1). As expected, GFP-positive cells isolated using both TREE- and RoT-based strategies were enriched for edited cells when compared to GFP-negative and unsorted cell populations. We found that base editing efficiency at these three target loci in HEK293 cells using RoT-based approaches was similar to those reported previously (Supplementary Table S8) (15). Importantly, this analysis also revealed across all three targeted sites that GFP-positive cells isolated via TREE had a statistically significant higher frequency of base editing than GFP-positive cells isolated using traditional RoT approaches.

Because of the success of targeting these loci, we investigated if TREE could be utilized to target additional genomic sites that display very low editing efficiency when traditional RoT approaches are applied. One such example is the *APOE* locus, a well-established risk factor associated with altered probability of sporadic Alzheimer's disease onset (25). Human *APOE* has three major isoforms, ApoE2, ApoE3 and ApoE4, which differ by two amino acid substitutions at positions 112 and 158 in exon 4—ApoE2 (Cys112, Cys158), ApoE3 (Cys112, Arg158), ApoE4 (Arg112, Arg158). Attempts to use base editing to convert ApoE3 to ApoE2 by targeting the APOE(R158) locus revealed undetectable levels of editing in unsorted cell populations despite similar transfection efficiencies when other genomic sites (Sites 1–3) were targeted (Supplementary Figure S2A). In addition, our attempts to use RoT-based methods in HEK293 cells to convert ApoE3 to ApoE2 by targeting the APOE(R158) locus revealed very low levels of editing in GFP+ isolated cells (Supplementary Figure S2B), further establishing the APOE(R158) locus as recalcitrant to genomic editing. We then used TREE-based methods to edit this same loci in HEK293 cells by co-transfecting pEF-BFP, pCMV-BE4-Gam and pDT-sgRNA with a sg(TS) targeting the APOE(R158) locus. As expected, flow cytometry analysis demonstrated that the transfection efficiency when TREE was used to target the APOE(R158) locus was similar to when TREE was used to target other genomic sites (Supplementary Figure S2C). In addition, despite these similarities in transfection efficiencies, there was no detectable editing in the unsorted cell populations using TREE to target the APOE(R158) locus, thereby confirming the difficulty in editing this genomic location (Supplementary Figure S2D). However, unlike in GFP-positive isolated using RoT methods, GFP-positive cells purified using TREE methods displayed a high level of base editing at the APOE(R158) locus (Supplementary Figure S2E). Together, these results demonstrate that TREE can not only provide for a higher level of enrichment of base-edited cell populations compared to conventional RoT strategies but also can allow for isolation of base-edited cells at genomic loci that were not previously achievable with traditional RoT approaches.

At last, we wanted to confirm that the fluorescent signal associated with cells isolated by TREE was transient. To that end, we measured the long-term fluorescence of GFP-positive cells purified after TREE-based editing (Supplementary Figure S3A). Notably, analysis of these cells by fluorescent microscopy (Supplementary Figure S3B) and flow cytometry (Supplementary Figure S3C) revealed no long-term detectable GFP signal, verifying that the TREE fluorescent output is indeed transient in nature.

### Multiplex base-editing using TREE

**Figure 4.**
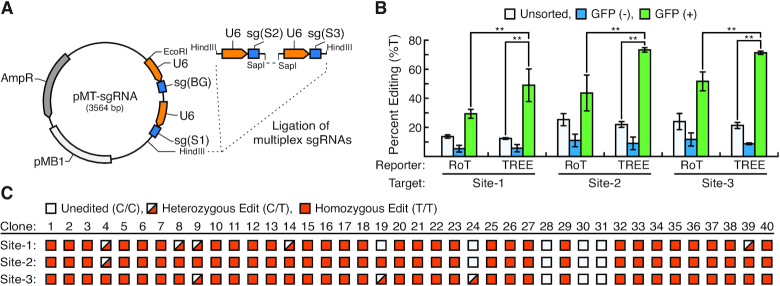
TREE enables efficient multiplex base editing. (**A**) Plasmid map of pMT-sgRNA vector that contains sg(BG) in addition to sgRNA for multiple target sites. Expression for all sgRNA cassettes is driven by separate U6 promoters (orange arrows). The HindIII restriction site allows for additional sgRNAs for target sites to be cloned in through restriction enzyme-based cloning. (**B**) Quantification of multiplex base editing efficiency at Site-1, Site-2 and Site-3 in GFP-positive, GFP-negative and unsorted cell populations using TREE- or RoT-based enrichment strategies. *n* = 3; * = *P* < 0.05, ** = *P* < 0.01. (**C**) Clonal analysis of editing at multiple genomic loci using TREE. 40 GFP-positive clones were isolated via single-cell sorting. Editing was detected via PCR and Sanger sequencing. Blank icon indicates no editing observed, half-red icon indicates heterozygous C and T at the target site, and solid red icon indicates homozygous T edits at the genomic site.

We further investigated if TREE could be utilized in conjunction with multiplexed genome engineering strategies. To accomplish this, we generated a multi-targeted vector (pMT-sgRNA) that contains sg(BG) as well as sgRNA for genomic targets Sites 1–3 (Figure 4A). In a similar manner to when TREE was employed to target a single locus, we utilized TREE to simultaneously target multiple genomic sites by co-transfecting HEK293 cells with pMT-sgRNA, pEF-BFP and pCMV-BE4-Gam. In parallel, we used a RoT-based approach by co-transfecting HEK293 cells with pMT-sgRNA, pEF-GFP and pCMV-BE4-Gam. After 48 h, GFP-negative and GFP-positive cells were isolated using flow cytometry (Supplementary Figure S4A). Along similar lines to single locus targeting, Sanger sequencing of the multiplex targeted genomic sites in GFP-positive cell populations isolated from TREE and RoT approaches revealed that TREE allowed for statistically significant higher frequency of base editing than RoT approaches (Figure 4B and Supplementary Figure S4B). Importantly, this analysis revealed that there was no statistically significant difference in editing efficiency when TREE was used to target these sites individually or a multiplexed manner (Supplementary Figure S4C). Finally, we wanted to determine if TREE increased the likelihood of C-to-T conversions at off-target loci. Therefore, in GFP-positive cell populations isolated from TREE and RoT approaches we PCR-amplified and Sanger sequenced the top predicted off-target loci for the sgRNA sequences used for multiplexed editing. Overall, quantification of the Sanger chromatographs by EditR revealed no observable C-to-T conversions at these off-target loci in either GFP-positive cells isolated with TREE- or RoT-based strategies when compared to that of untransfected cells (Supplementary Figure S5).

Sanger sequencing that was performed on bulk sorted GFP-positive cells suggested that multiplex editing in conjunction with TREE could result in multiplexed editing in the same cell. To confirm that this indeed occurred, we again used our multi-targeting vector (pMT-sgRNA) in conjunction with TREE to simultaneously target genomic Sites 1–3 in HEK293 cells. We then sorted single GFP-positive cells into a 96-well plate. After expansion, Sanger sequencing of the multiplexed genomic sites was performed on a total of 40 clones. This analysis revealed that 36 out of the 40 clones had base editing at more than one genomic site (Figure 4C). Remarkably, this analysis revealed that almost 80% of the isolated clones (31 out of 40) had biallelic conversions at all three genomic loci.

One of the caveats of all base-editing approaches, regardless if RoT- or TREE-based enrichment strategies are employed, is that base editors can potentially edit non-target Cs that are located in an 6 nt window (termed the editing window) within the protospacer (26). As a consequence, this could potentially limit the application of base editing approaches in which conversion of non-target Cs result in a non-silent mutation or other phenotypic changes. To that end, we wanted to determine if any of our clones contained edits exclusively at the target C and not any other Cs within the editing window. Indeed, we identified a number of clones in which at genomic Site 2 and Site 3 modification only occurred at the target C (Supplementary Figure S6). Interestingly, we did not identify any clones in which at genomic Site 1 such exclusive modification of the target C occurred. We speculate that because another C occurs immediately adjacent to this target C, that such exclusive modification will require the use of recently published site-specific editors that allow for single nucleotide changes free from off-targeting conversions within the editing window (26).

### TREE allows for highly efficient editing in human pluripotent stem cells (hPSCs)

**Figure 5.**
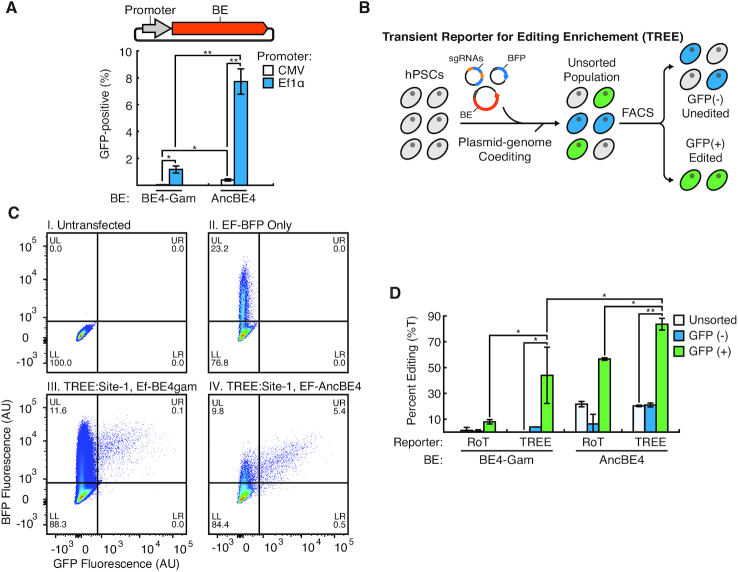
Highly efficient editing in human pluripotent stem cells (hPSCs) using TREE (**A**) Quantification of base editing efficiency (percentage GFP-positive cells) when hPSCs were co-transfected with pEF-BFP, sg(BG) and various base editing vectors. *n* = 3; * = *P* < 0.05, ** = *P* < 0.01. (**B**) Schematic for enrichment of edited hPSC using TREE. HPSCs were co-transfected with pEF-BFP, pEF-BE4-Gam/pEF-AncBE4 and pDT-sgRNA vectors. 48 h post-transfection, flow cytometry was used to sort cell populations into GFP-positive and -negative fractions. (**C**) Representative flow cytometry plots of (i) untransfected hPSCs cells and (ii) hPSCs transfected with pEF-BFP only as well as hPSCs cells in which TREE was applied targeting Site-1 utilizing (iii) pEF-BE4-Gam or (iv) pEF-AncBE4. (**D**) Quantification of base editing efficiency at Site-1 in GFP-positive, GFP-negative and unsorted cell populations isolated using TREE- or RoT-based enrichment strategies in which pEF-BE4-Gam or pEF-AncBE4 was employed. *n* = 3; * = *P* < 0.05, ** = *P* < 0.01.

Single base pair modification in hPSCs via CRISPR/Cas9-induced DSB followed by HDR suffers from low efficiencies (5–9). In addition, genomic modification of hPSCs using deaminase-based DNA base editor has yet to be reported. Therefore, we wanted to investigate if TREE could be utilized to efficiently edit specific loci in hPSCs. Hence, we co-transfected pEF-BFP and pCMV-BE4-Gam into hPSCs using a transfection reagent (Lipofectamine™ Stem) that had been previously used by others for the efficient delivery of Cas9-related plasmids to hPSCs (27,28). Surprisingly, we did not observe many GFP-positive cells in these cell populations (Figure 5A and Supplementary Figure S7A). As such, we performed similar experiments in which we employed a more recently published, higher efficiency base editor, AncBE4max (herein referred to AncBE4) (29). Briefly, AncBE4 is an improved version of BE4 that has been codon optimized for expression and contains an ancestral reconstructed deaminase to increase base editing efficiency at target loci. Nonetheless, similar to when BE4-Gam was utilized, we observed very few GFP-positive cells when AncBE4 was used (Supplementary Figure S7A). Because previous reports have suggested that the CMV promoter is inefficient for transgene expression in pluripotent stem cells (30–32), we replaced the promoter driving base editor expression with EF1α. When hPSCs were co-transfected with pEF-BE4-Gam or pEF-AncBE4 as well as pEF-BFP and sg(BG), a significant number of GFP-positive cells were observed (Figure 5A). Using the pEF-AncBE4 vector, we also optimized editing efficiency in hPSCs by using a range of base editor amount at varying ratio with sg(BG) (Supplementary Figure S7B). Similar to our optimization experiments with HEK293 cells, this analysis revealed that base editing efficiencies were significantly affected by these parameters. Interestingly, the most optimal parameters in hPSCs differed from those identified in HEK293 cells (Figure 2D) highlighting the utility of this assay to evaluate these variables. Using these optimized base editing vector designs, we applied TREE to target a genomic loci in hPSCs by co-transfecting pEF-BE4-Gam/pEF-AncBE4, pEF-BFP and pDT-sgRNA (with a sg(TS) targeting site 1) (Figure 5B). In turn, flow cytometry was used to isolate GFP-positive and -negative cell populations (Figure 5C). Subsequently, Sanger sequencing was performed on the targeted genomic site in GFP-positive, GFP-negative and unsorted cell populations isolated from TREE and RoT approaches in which pEF-BE4-Gam and pEF-AncBE4 was used (Supplementary Figure S7C). This analysis demonstrated that GFP-positive hPSCs isolated via TREE had a statistically significant higher frequency of base editing than GFP-positive hPSCs isolated using traditional RoT approaches (Figure 5D). In addition, TREE employed with the pEF-AncBE4 vector allowed for the efficient modification of the difficult to edit APOE(R158) locus (Supplementary Figure S7D and E).

Similar to our work with HEK293 cells, we wanted to confirm that the fluorescent output of TREE was transient in nature. In that regard, we measured the fluorescence of GFP-positive hPSCs isolated after TREE-based editing. Flow cytometry analysis revealed that after 2 weeks of culture there was no detectable GFP signal (Supplementary Figure S8), demonstrating that the fluorescent signal associated with hPSCs purified by TREE was transient.

Collectively, although this data demonstrates that TREE can be utilized for the efficient base editing of hPSCs, one of the caveats of all base editing approaches is the C-to-T conversion of non-target Cs within the editing window (26,33). Indeed, the Sanger sequencing analysis of GFP-positive populations isolated from TREE revealed editing of such non-target Cs when either Site 1 (Supplementary Figure S7C) or the APOE(R158) (Supplementary Figure S7E) locus was targeted in hPSCs. As such, to determine whether TREE allowed allelic outcomes in which targeting only occurred at the desired C, we performed NGS of PCR amplicons of Site 1 and APOE(R158) in GFP-positive cells purified using TREE. This analysis revealed at both these loci a very modest number of allelic outcomes in which base editing occurred exclusively at the target C, free from confounding C-to-T conversions at other sites within the targeting window (Supplementary Figure S9). Instead, the most common editing outcome was one in which the majority of the Cs in the editing window were converted to Ts (Supplementary Figure S9). This suggests that for future applications which require a higher percentage of allelic outcomes where editing occurs only at the target C the use of recently published base editors that have a narrower editing window (26,33) will be required. Nonetheless, this collective data demonstrates the broad utility of TREE to allow for the efficient editing in hPSCs.

## DISCUSSION

Since the first deaminase base editor was developed by Komor *et al.* (11), multiple additional base-editing technologies have been rapidly developed with various endonucleases, deaminases, targeting windows and PAM specificities (13). Application of these emerging base editors to new cell types requires a slow, iterative process in which various base editing parameters are tested and editing efficiency is assessed through downstream sequencing methods. Additionally, as we demonstrate, transfection efficiency does not precisely correlate with editing efficiency, so reporters of transfection do not provide accurate information about the efficacy of various base editing strategies. Here, we describe how BFP-to-GFP conversion and TREE can be utilized to rapidly optimize various factors that influence base editing efficiency, including base editor plasmid concentration and design as well as base editor to sgRNA ratios. In fact, we show that these parameters are cell line-specific, demonstrating the advantage of TREE to allow for the high-throughput evaluation of base editing approaches. In the future, we can utilize TREE in the context of high-throughput screening to identify small molecules to further enhance base editing efficiency in a manner similar to that which has been previously achieved with CRISPR-mediated HDR approaches (34,35).

It has been shown that CRISPR/Cas9 genome engineering is compatible with a variety of delivery methods (e.g. lipid-mediated transfection, electroporation) and expression systems (e.g. plasmid DNA, Cas9-gRNA ribonucleoprotein complexes [RNP]), each with advantages and disadvantages that have been reviewed extensively elsewhere (36,37). In this study, we employed lipid-based delivery reagents that have been previously employed by others for the CRISPR/Cas9-based editing of HEK293 cells (Lipofectamine 3000; 38) and hPSCs (Lipofectamine Stem; 24). Given TREE’s ease of use and readily detectable fluorescent output we anticipate that TREE can be employed with whatever transfection method that is preferred by the end user. For instance, we demonstrated that our base editing assay was compatible with both plasmid and RNP approaches. Although we observed that the overall genomic base editing efficiency of RNP-based expression was lower than that of lipid-based expression, we provide proof-of-principle that TREE can be employed in future applications where the advantages of RNPs are desirable.

One potential limitation of the use of the plasmid DNA expression systems in the context of TREE approaches is random integration of all or part of the plasmid DNA into the genome of targeted cells. It should be noted that it has been reported by others that the stable integration of circular plasmid DNA into the host genome is infrequent, especially for cells such as hPSCs where it has been reported on the order of 1 per 1 × 10^5^ cells (39–41). Indeed, as it relates to potential integration of the pEF-BFP plasmid, we demonstrate that the fluorescent output of TREE is transient in both HEK293 cells and hPSCs, suggesting that this plasmid does not integrate into the genome. As it relates to the integration of the base editing and sgRNA plasmids, it has been shown by others in CRISPR/Cas9 genome engineering that the Cas9 and sgRNA plasmids can be integrated at on- and off-target sites (23). However, we speculate that because base editors do not introduce DSBs the integration of these plasmids into the genome would be infrequent. In fact, we did not observe any integration of these plasmids when Sanger sequencing or NGS was performed at the on- or off-target sites. Moving forward, undesirable insertions of plasmid DNA sequences at target sites can be detected using PCR-based methods followed by Sanger sequencing or NGS of the resultant amplicons. On the other hand, similar insertions at off-target or random genomic sites are difficult to detect and will require the use of more comprehensive techniques such as whole genome sequencing.

Human cell models are critical for elucidating the mechanisms of disease progression as well as identifying and testing potential therapeutic interventions. Because a high percentage of human diseases are due to single nucleotide polymorphisms (SNPs) (42), base editors can allow for the precise engineering of *in vitro* models of human disease. Here we provide proof-of-principle that TREE can be employed to edit disease-relevant loci. Specifically, we demonstrate that TREE enables for the enrichment of cells that had been edited at the APOE(R158) locus, a gene associated with altered risk of Alzheimer's disease onset (25). Notably, conventional RoT-based methods did not allow for significant enrichment of edited cells at this same refractory locus. In addition, because many human diseases are multigenetic disorders that are a result of complex gene interactions, we also investigated the ability of TREE to be utilized in multiplexed genome engineering applications. By using a multi-targeted vector, we demonstrated that compared to RoT-based methods TREE resulted in a significantly higher level of cells enriched for simultaneous editing at multiple independent loci. In fact, we demonstrated that through analysis of single cell clones that 90% of the clones had simultaneous base editing at more than one genomic site and almost 80% of the clones had biallelic conversions at all three targeted loci. In this vein, TREE provides a highly efficient method for generating cell-based models of multigenic diseases.

Many immortalized cell lines, such as HEK293s, are aneuploid with unknown mutations and dosage at key disease-relevant genes. Alternatively, hPSCs, which have a normal euploid karyotype and the potential to differentiate into all cell types of the mature adult body, represent an attractive alternative to immortalized cell lines for disease modeling and drug screening applications (43–46). In particular, the ability to use gene editing technologies to generate isogenic hPSC lines that differ only with respect to disease mutations has great potential as it relates to precisely defining genotype to phenotype relationships (34). The RNA-guided CRISPR-Cas9 system has the potential to allow for precise genetic modifications in hPSCs through the introduction of site-specific DSBs. Although previous reports demonstrate that introduction of DSB via CRISPR/Cas9 significantly improves the ability to obtain knock out cell lines from hPSCs by the NHEJ pathway (9), single base modification using CRISPR/Cas9-induced DSB followed by HDR is extremely inefficient (1–2% of sequenced colonies in which one allele is targeted and <1% where both alleles are targeted; (5–9). Recently, it has been reported that co-delivery of Cas9, sgRNA, and a puromycin selection cassette followed by transient puromycin selection can increase the HDR-mediated genome engineering in hPSCs (47,48) However, these strategies rely on the introduction of DSBs, which in pluripotent stem cells can lead to large deletions and complex chromosomal rearrangements (49), significant cytotoxicity (50) and increased acquisition of p53 mutations (51). In addition, it has been shown that the use of antibiotic selection, even in a transient manner, may lead to the selection of hPSCs, with chromosomal abnormalities (52,53). Yet, to our knowledge, base editors, which do not have these same limitations as CRISPR/Cas9-induced DSB followed by HDR, have not previously been used with hPSCs. In fact, our initial attempts to apply base editors in the context of both RoT- and TREE-based approaches with hPSCs did not allow for observable modification of target loci. Instead, by replacing the standard CMV promoter in the base editing plasmids with an EF1α promoter, we were able to achieve modification of genomic sites using both RoT- and TREE-centered methods. However, TREE allowed for significantly higher enrichment of edited hPSCs when compared to RoT isolation strategies. We contend that the use of TREE with hPSCs will significantly advance the use of these cells in disease modeling, drug screening, and cell-based therapies.

Despite their tremendous potential in a variety of downstream applications, base editing approaches have a few of caveats that should be noted, regardless of whether RoT- or TREE-based enrichment strategies are employed. First, as is the case with all Cas9-directed genome editing approaches, is the potential for genome modification at off-target loci (19,54). In this work, GFP-positive cells isolated via TREE did not display untargeted C-to-T conversions at the off-target genomic loci examined. Recently, it has been reported that base editors can induce site-specific inosine formation on RNA (55). Accordingly, in the future, the effect of TREE-based approaches on unwanted RNA modifications should be examined. Another limitation of base editing methods is modification of additional C nucleotides that are in close proximity to the target C (26). In fact, some base editors can cause C-to-T conversions at any Cs in up to a 9-nt window within the protospacer (26,11,56,57). Such C-to-T modifications could be especially problematic if they result in amino acid alterations during translation, induce epigenetic changes or cause other phenotypic changes in targeted cells. To that end, through clonal isolation and next generation sequencing (NGS) analysis we identified that such exclusive modifications of the target C were achieved in both edited HEK293 cells or hPSCs that were enriched using TREE-based methods. It should be noted, though, that at genomic Site-1, where a C lies adjacent to the target C, allelic outcomes in which modification only occurred at the target C were rare events. Moving forward, modified base editors that have a narrow editing window (33,26) could be easily employed with TREE to target such genomic loci that contain multiple Cs in close proximity to the target C.

In summary, we demonstrate that TREE allows not only for the optimization of base editing strategies in the context of a variety of cell types and genomic locations but also the enrichment of cell populations to be utilized in variety of downstream applications. In particular, with the rate at which the genome editing field has been progressing over the past few years, TREE is a readily adoptable method that will expedite and improve tractability of single-nucleotide genome engineering methods.

## Supplementary Material

gkz713_Supplemental_File
