# A transient reporter for editing enrichment (TREE) in human cells

**DOI:** 10.1093/nar/gkaa027

**Published:** 2020-01-16

**Authors:** Kylie Standage-Beier, Stefan J Tekel, Nicholas Brookhouser, Grace Schwarz, Toan Nguyen, Xiao Wang, David A Brafman

**Affiliations:** 1 School of Biological and Health Systems Engineering, Arizona State University, Tempe, AZ 85287, USA; 2 Molecular and Cellular Biology graduate program, Arizona State University, Tempe, AZ 85287, USA; 3 Graduate Program in Clinical Translational Sciences, University of Arizona College of Medicine-Phoenix, Phoenix, AZ 85004, USA

*Nucleic Acids Research*, 2019, 47(19): e120, https://doi.org/10.1093/nar/gkz713

The reference to a recent article has been added to the Introduction:

… Some progress has been made to help enrich for edited cells, such as co-transfecting plasmids with a fluorescent reporter and using flow cytometry to isolate reporter-positive cells. Similarly, base editors fused to fluorescent proteins have been used to enrich for edited cell populations (16,**58**)…

58. Coelho M.A., Li S., Pane L.S., Firth M., Ciotta G., Wrigley J.D., Cuomo M.E., Maresca M., Taylor B.J.M. (2018) BE-FLARE: a fluorescent reporter of base editing activity reveals editing characteristics of APOBEC3A and APOBEC3B. *BMC Biol*. **16**:150.

This addition does not affect the results and conclusion of the study.

May 2020: an additional correction was made to remove the sentence “However, these techniques are only reporters of transfection (RoT) and do not report on base editing activity within a cell population." from the introduction. This change has been made online but is not reflected in the print version of either the original article (https://doi.org/10.1093/nar/gkz713) or the corrigendum (https://doi.org/10.1093/nar/gkaa027).

