# Supplementary material for: A transient reporter for editing enrichment (TREE) in human cells
Source: Nucleic Acids Res. 2019 Aug 20;47(19):e120. doi: 10.1093/nar/gkz713 (PMC6821290; doi:10.1093/nar/gkz713)
Supplement: gkz713_Supplemental_File [file gkz713_supplemental_file.pdf]

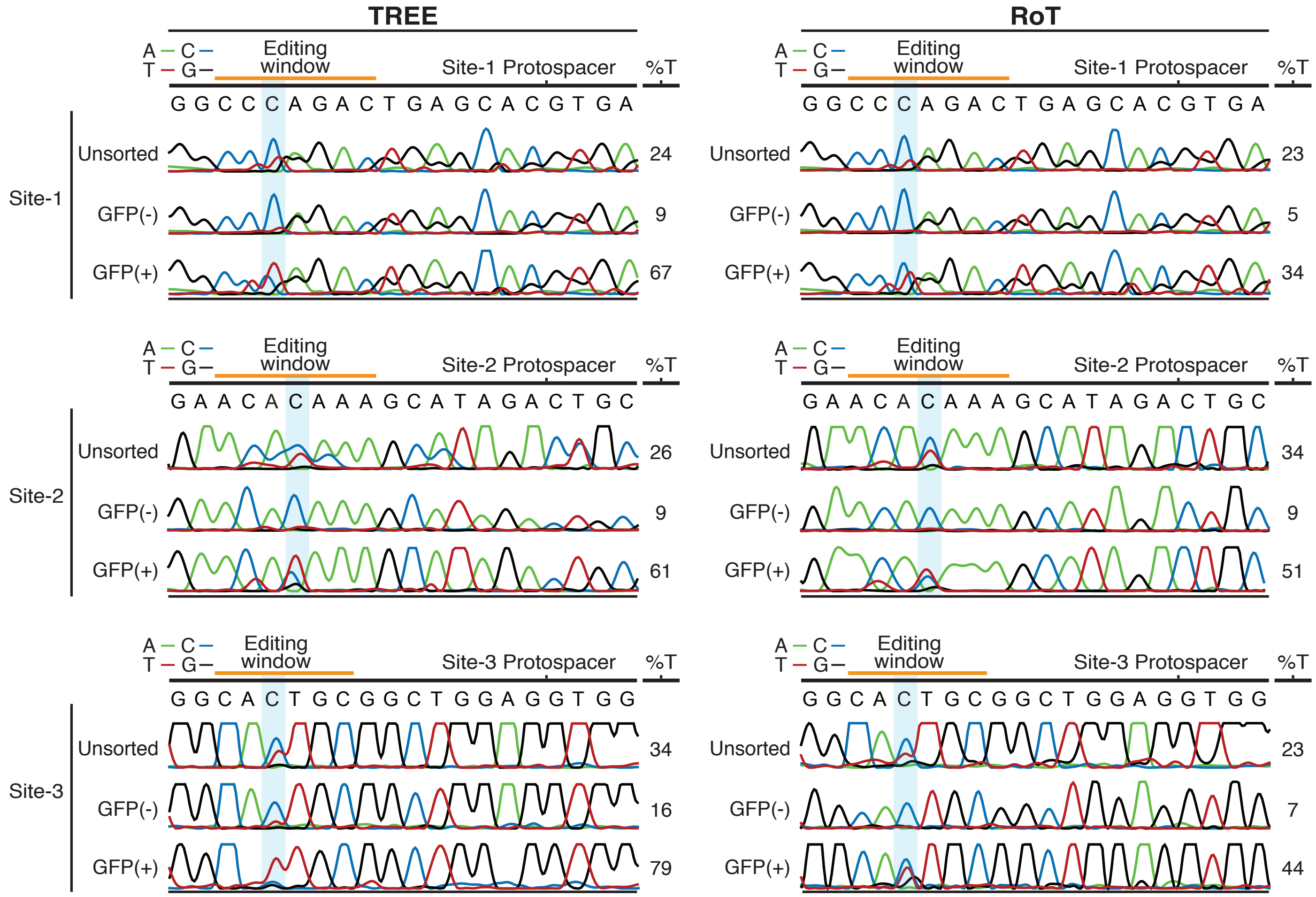

Supplementary Figure 1. Representative Sanger sequencing chromatographs of edited HEK293 cells enriched using TREE- and RoT-based approaches. Sanger sequencing chromatographs of Site-1, Site-2, and Site-3 of GFP-positive, GFP-negative, and unsorted cell populations isolated with TREE- and RoT-based approaches.

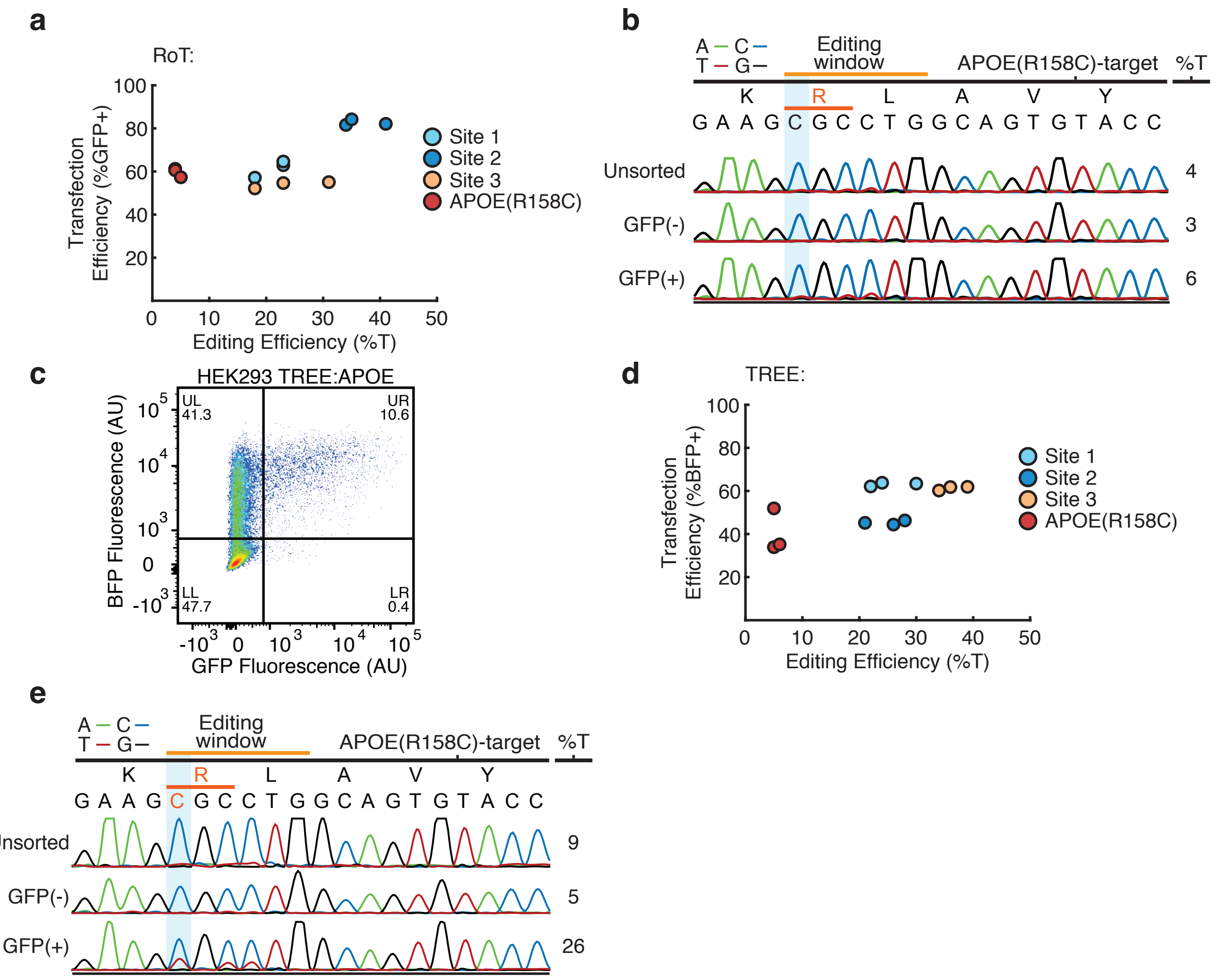

Supplementary Figure 2. TREE allows for base editing of refractory APOE(R158) locus in HEK293 cells. (**a**) HEK293 cells were transfected with pEF-GFP, pCMV-BE4-Gam, and sg(TS). Comparison of transfection efficiency (percentage of GFP-positive cells) and editing efficiency (percentage of C-to-T conversion at target nucleotide) in unsorted cell populations at Site-1, Site-2, Site-2, and APOE(R158) locus. (**b**) Representative Sanger sequencing chromatographs of APOE(R158) locus in GFP-positive, GFP-negative, and unsorted cell populations isolated with RoT-based methods. (**c**) Representative flow cytometry plot of HEK293 cells in which TREE was applied targeting the APOE(R158) locus. (**d**) HEK293 cells were transfected with pEF-BFP, pCMV-BE4-Gam, and pDT-sgRNA. Comparison of transfection efficiency (percentage of BFP-positive cells) and editing efficiency (percentage of C-to-T conversion at target nucleotide) in unsorted cell populations at Site-1, Site-2, Site-3, and APOE(R158) locus. (**e**) Representative Sanger sequencing chromatographs of APOE(R158) locus in GFP-positive, GFP-negative, and unsorted cell populations isolated with TREE-based methods.

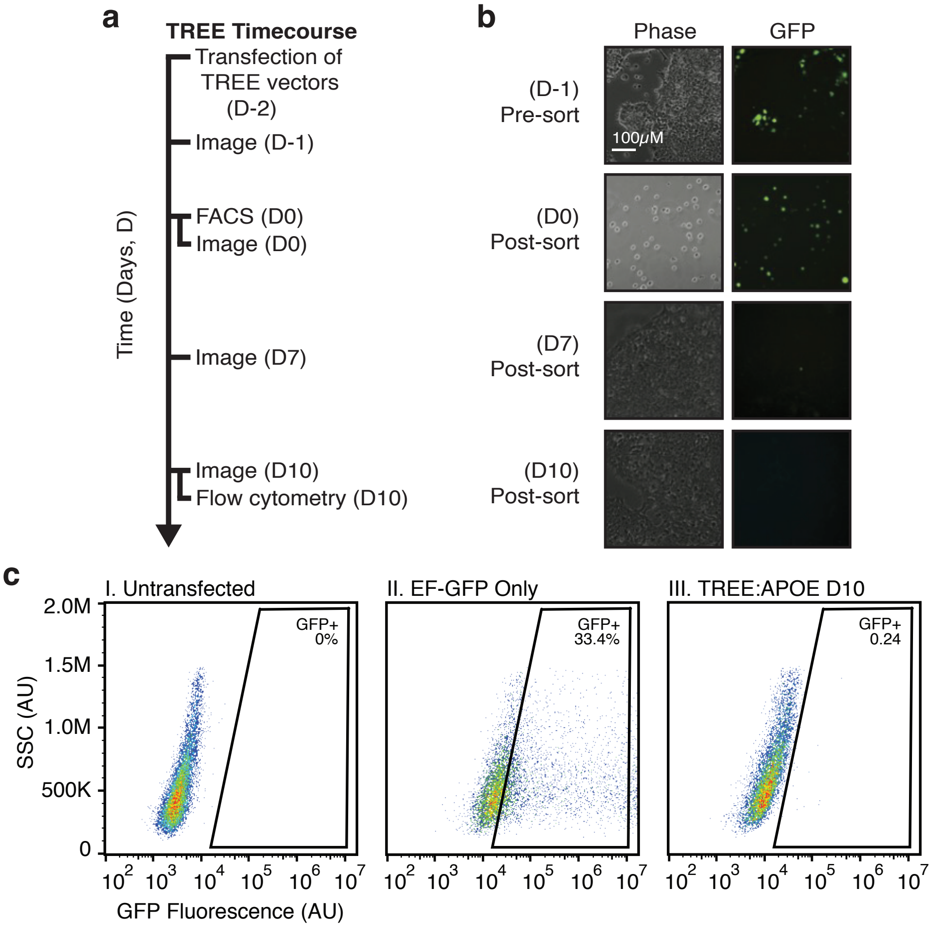

Supplementary Figure 3. TREE fluorescent output in HEK293 cells is transient. (**a**) HEK293 cells were transfected with pEF-BFP, pCMV-BE4-Gam, and pDT-sgRNA and GFP-positive cells were isolated by flow cytometry. Replated GFP-positive cells were analyzed by fluorescent microscopy and flow cytometry at various time points post-sorting. (**b**) Representative fluorescent microscopy images of cells prior to cell sorting (D-1, Pre-sort) and various time points (D0, D7, D10) after sorting. (**c**) Representative flow cytometry plots of (i) untransfected HEK293 cells, (ii) pEF-GFP transfected HEK293 cells, and (iii) TREE-enriched GFP-positive HEK293 cells 10 days after sorting.

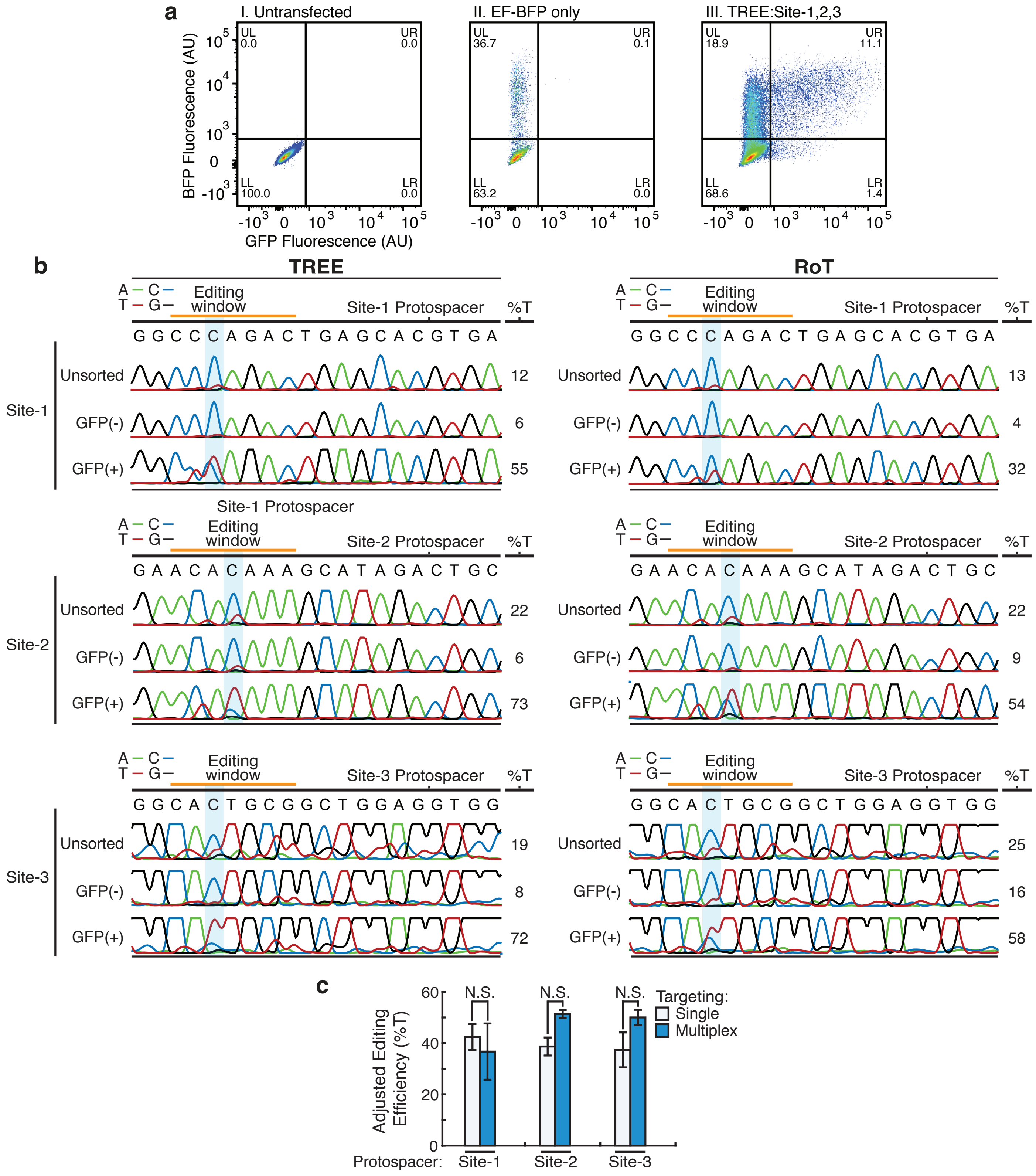

Supplementary Figure 4. Analysis of multiplexed edited HEK293 cells using TREE- and RoT-based methods. (**a**) Representative flow cytometry plot of HEK293 cells in which multiplex TREE was applied simultaneously targeting Site-1, Site-2, and Site-3. (**b**) Representative Sanger sequencing chromatographs of the Site-1, Site-2, and Site-3 loci in GFP-positive, GFP-negative, and unsorted cell populations isolated with TREE multiplex-based methods. (**c**) Comparison of base editing efficiencies at Site-1, Site-2, and Site-3 in GFP-positive, GFP-negative, and unsorted cell populations using TREE-based methods to target these sites individually or in a multiplexed manner. n=3; N.S. = not significant.

**BG-OT1:**	C	C	C	T	A	C	A	T	C	G	T	G	C	A	G	T	G	C	T	T
**Untransfected:**			94			95														
**TREE:**			95			95														
**BG-OT2:**	C	C	C	C	A	A	G	T	A	G	T	G	C	A	G	T	G	C	T	T
**Untransfected:**			97	90																
**TREE:**			97	94																
**BG-OT3:**	A	A	C	C	A	A	G	A	T	G	T	G	C	A	G	T	G	C	T	T
**Untransfected:**			95	93																
**TREE:**			91	96																
**BG-OT4:**	A	A	C	C	A	G	C	G	C	C	T	G	C	A	G	T	G	C	T	T
**Untransfected:**			95	96			96													
**TREE:**			95	97			95													
**BG-OT5:**	C	C	C	C	A	T	G	G	C	T	T	G	C	T	G	T	G	C	T	T
**Untransfected:**			97	91																
**TREE:**			98	96																
**Site1-OT1:**	C	A	C	C	C	A	G	A	C	T	G	A	G	C	A	C	G	T	G	C
**Untransfected:**			98	97	98															
**RoT:**			97	98	98															
**TREE:**			98	96	97															
**Site1-OT2:**	G	A	C	A	C	A	G	A	C	C	G	G	G	C	A	C	G	T	G	A
**Untransfected:**			98		95															
**RoT:**			98		97															
**TREE:**			98		97															
**Site1-OT3:**	A	G	C	T	C	A	G	A	C	T	G	A	G	C	A	A	G	T	G	A
**Untransfected:**			98		98															
**RoT:**			97		97															
**TREE:**			89		97															
**Site1-OT4:**	A	G	A	C	C	A	G	A	C	T	G	A	G	C	A	A	G	A	G	A
**Untransfected:**				96	96															
**RoT:**				94	96															
**TREE:**				92	93															
**Site1-OT5:**	G	A	G	C	C	A	G	A	A	T	G	A	G	C	A	C	G	T	G	A
**Untransfected:**				96	95															
**RoT:**				96	95															
**TREE:**				95	94															
**Site2-OT1:**	G	A	A	C	A	C	A	A	T	G	C	A	T	A	G	A	T	T	G	C
**Untransfected:**				80		92														
**RoT:**				97		96														
**TREE:**				94		94														
**Site2-OT2:**	G	C	A	G	T	C	T	A	T	G	C	T	T	T	A	T	G	T	T	T
**Untransfected:**						90														
**RoT:**						88														
**TREE:**						89														

Continued on next page.

**Site3-OT1:**	T	G	C	A	C	T	G	C	G	G	C	C	G	G	A	G	G	A	G	G
**Untransfected:**			91		94			92												
**RoT:**			93		93			93												
**TREE:**			89		90			92												

**Site3-OT2:**	G	G	C	T	C	T	G	C	G	G	C	T	G	G	A	G	G	G	G	G
**Untransfected:**			90		84			93												
**RoT:**			95		91			95												
**TREE:**			94		82			94												

**Site3-OT3:**	G	G	C	A	C	G	A	C	G	G	C	T	G	G	A	G	G	T	G	G
**Untransfected:**			92		96			95												
**RoT:**			93		88			93												
**TREE:**			91		91			93												

**Site3-OT4:**	G	G	C	A	T	C	A	C	G	G	C	T	G	G	A	G	G	T	G	G
**Untransfected:**			92			97		96												
**RoT:**			94			90		94												
**TREE:**			94			91		92												

**Site3-OT4:**	G	G	C	G	C	T	G	C	G	G	C	G	G	G	A	G	G	T	G	G
**Untransfected:**			98		94			95												
**RoT:**			96		94			98												
**TREE:**			97		82			98												

Supplementary Figure 5. Analysis of off-target sites in multiplexed edited HEK293 cells using TREE- and RoT-based methods. GFP-positive cell populations isolated from TREE and RoT approaches were PCR-amplified and subjected to Sanger sequencing on the top predicted off-target loci for the sgRNA sequences for sg(BG) and genomic Sites 1-3. The C nucleotides in red text are potential Cs that can undergo C-to-T conversion within the editing window in the protospacer. The numbers below each C are quantification of the percentage of Cs of the Sanger sequence chromatograms using EditR.

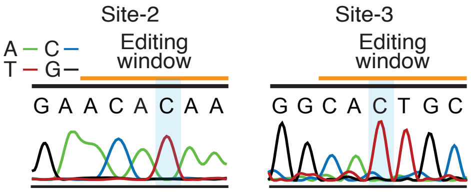

Supplementary Figure 6. Identification of exclusive targeting events in clonal population in edited HEK293 cells using TREE. Representative Sanger sequencing chromatographs of clonal cell populations that contain edits exclusively at the target C and not any other Cs within the editing window.

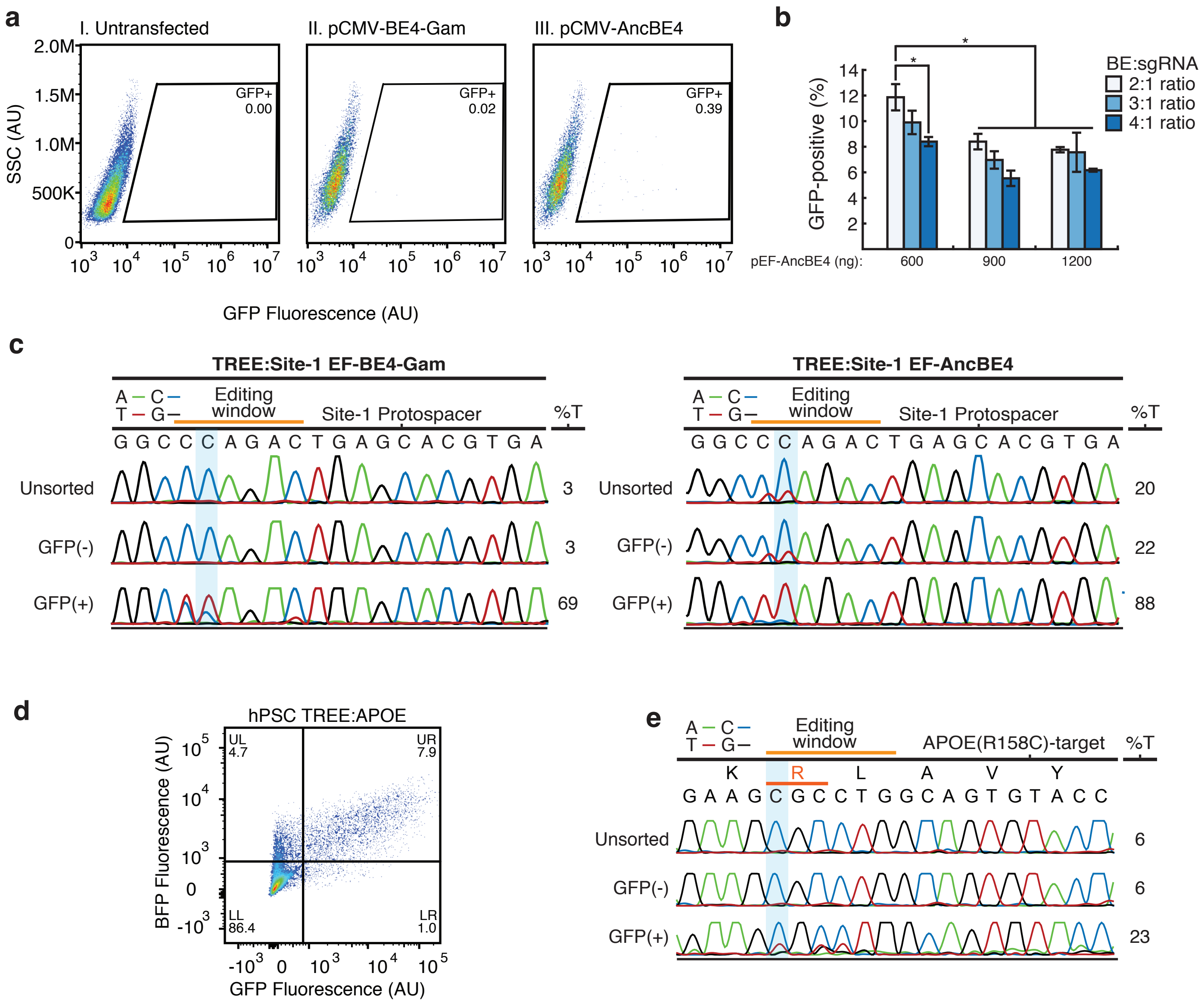

Supplementary Figure 7. TREE allows for base editing in hPSCs. (**a**) Representative flow cytometry plots in which TREE was employed in hPSCs utilizing (i) untransfected (ii) pCMV-BE4-Gam or (iii) pCMV-AncBE4. (**b**) Editing efficiency (percentage GFP-positive cells) of targeting in hPSCs line with various amounts of pEF-AncBE4 plasmid and ratios with the sg(BG) vector. n = 3, * = p<0.05. (**c**) Representative Sanger sequencing chromatographs of Site-1 in GFP-positive, GFP-negative, and unsorted cell populations isolated with TREE- and RoT-based methods in which pEF-BE4-Gam or pEF-AncBE4 was utilized. (d) Representative flow cytometry plot of hPSCs in which TREE was applied targeting the APOE(R158) locus. (**e**) Representative Sanger sequencing chromatographs of APOE(R158) locus in GFP-positive, GFP-negative, and unsorted cell populations isolated with TREE-based methods.

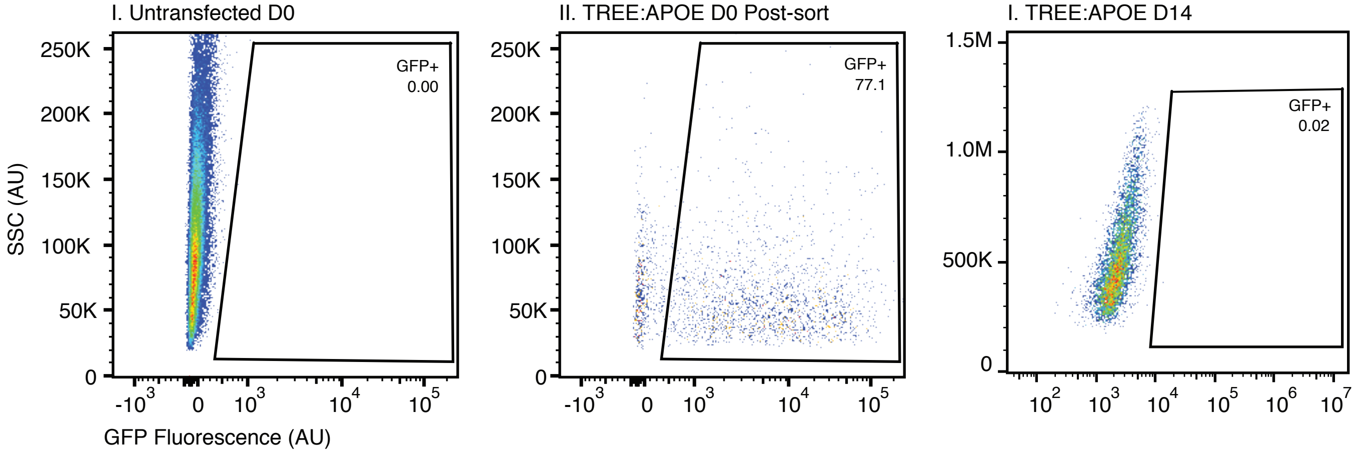

Supplementary Figure 8. TREE fluorescent output in hPSCs is transient. Representative flow cytometry plots of (i) untransfected hPSCs, (ii) TREE-enriched GFP-positive hPSCs 0 days (iii) 14 days after sorting.

a

	Untransfected Site-1 (Editing window, PAM)	% of Reads	TREE:Site-1 (Editing window, PAM)	% of Reads
WT	GGCCCAGACTGAGCACGTGATGG	99.7	GGCCCAGACTGAGCACGTGATGG	5.2
18	GGTCCAGACTGAGCACGTGATGG	0.1	GGTCCAGACTGAGCACGTGATGG	0.0
17	GGCTCAGACTGAGCACGTGATGG	0.1	GGCTCAGACTGAGCACGTGATGG	0.1
16	GGCCTAGACTGAGCACGTGATGG	0.1	GGCCTAGACTGAGCACGTGATGG	0.3
12	GGCCCAGATTGAGCACGTGATGG	0.0	GGCCCAGATTGAGCACGTGATGG	0.0
18,17	GGTTCAGACTGAGCACGTGATGG	0.0	GGTTCAGACTGAGCACGTGATGG	0.0
18,16	GGTCTAGACTGAGCACGTGATGG	0.0	GGTCTAGACTGAGCACGTGATGG	0.0
18,12	GGTCCAGATTGAGCACGTGATGG	0.0	GGTCCAGATTGAGCACGTGATGG	0.0
17,16	GGCTTAGACTGAGCACGTGATGG	0.0	GGCTTAGACTGAGCACGTGATGG	85.2
17,12	GGCTCAGATTGAGCACGTGATGG	0.0	GGCTCAGATTGAGCACGTGATGG	0.1
16,12	GGCCTAGATTGAGCACGTGATGG	0.0	GGCCTAGATTGAGCACGTGATGG	0.2
18,17,16	GGTTTAGACTGAGCACGTGATGG	0.0	GGTTTAGACTGAGCACGTGATGG	6.2
17,16,12	GGCTTAGATTGAGCACGTGATGG	0.0	GGCTTAGATTGAGCACGTGATGG	2.4
18,16,12	GGTCTAGATTGAGCACGTGATGG	0.0	GGTCTAGATTGAGCACGTGATGG	0.0
18,17,12	GGTTCAGATTGAGCACGTGATGG	0.0	GGTTCAGATTGAGCACGTGATGG	0.0
18,17,16,12	GGTTTAGATTGAGCACGTGATGG	0.0	GGTTTAGATTGAGCACGTGATGG	0.1

b

	Untransfected APOE (Editing window, PAM)	% of Reads	TREE:APOE (Editing window, PAM)	% of Reads
WT	GAAGCGCCTGGCAGTGTACCAGG	93.4	GAAGCGCCTGGCAGTGTACCAGG	64.2
16	GAAGTGCCTGGCAGTGTACCAGG	2.5	GAAGTGCCTGGCAGTGTACCAGG	5.2
14	GAAGCGTCTGGCAGTGTACCAGG	0.1	GAAGCGTCTGGCAGTGTACCAGG	1.1
13	GAAGCGCTTGGCAGTGTACCAGG	0.1	GAAGCGCTTGGCAGTGTACCAGG	1.0
16,14	GAAGTGTCTGGCAGTGTACCAGG	1.1	GAAGTGTCTGGCAGTGTACCAGG	1.2
16,13	GAAGTGCTTGGCAGTGTACCAGG	0.4	GAAGTGCTTGGCAGTGTACCAGG	1.0
14,13	GAAGCGTTTGGCAGTGTACCAGG	0.5	GAAGCGTTTGGCAGTGTACCAGG	7.0
16,14,13	GAAGTGTTTGGCAGTGTACCAGG	1.9	GAAGTGTTTGGCAGTGTACCAGG	19.2

Supplementary Figure 9. Next generation sequencing (NGS) analysis of allelic outcomes at target sites in hPSCs. NGS analysis for the target site when TREE was applied to edit Site-1 or the APOE(R158) in hPSCs. The number to left of the allelic outcome indicates the position upstream (5') relative to the PAM. Abbreviation: WT = wild-type unedited locus.

Supplementary Table 1. List of sgRNA sequences used in this study.

Site	Sequence (5'→3')
Site-1	GGCCCAGACTGAGCACGTGA
Site-2	GAACACAAAGCATAGACTGC
Site-3	GGCACTGCGGCTGGAGGTGG
APOE(R158)	GAAGCGCCTGGCAGTGTACC
BFP(H66Y)	GACCCACGGCGTGCAGTGCTT
C1ORF228	GTGCTGTTAGCACCCTGGAAA

Supplementary Table 2. List of primers used in this study to amplify on-target sites.

Primer	Forward Sequence (5'→3')	Reverse Sequence (5'→3')
Site-1	ATGTGGGCTGCCTAGAAAGG	CCCAGCCAAACTTGTCAACC
Site-2	CCAGCCCCATCTGTCAAACT	TGAATGGATTCCTTGGAAACAATGA
Site-3	TGGTCTTCTTTCCCCTCCCCTGCCCTCC	GGCCTGGAGGCGGGGGCTCAGAGA
APOE(R158)	GGACGAGACCATGAAGGAGTTGAAGGC	CCACCTGCTCCTTCACCTCGTCCAG

Supplementary Table 3. Parameters for EditR analysis.

Target Site	Sequencing Direction	Protospacer	5’ bound	3’ bound
Site-1	Forward	GGCCCAGACTGAGCACGTGA	GGCCTGGGTCAA	TTCCTTTCCTCTG
	Reverse	TCACGTGCTCAGTCTGGGCC	GAGGAAAGGAAGCCCTGCT	CAGGCCAGGGCTGGA
Site-2	Forward	GAACACAAAGCATAGACTGC	CCCGCTGGCCCTGT	TCAGGCTGGCCCGC
	Reverse	GCAGTCTATGCTTTGTGTTC	CCAGCCCGCTGGCCCTGTA	AGCTATTCAGGCT
Site-3	Forward	GTGGCACTGCGGCTGGAGGT	GATGACAGGCAGGGGCA	CAGCACCAGA
	Reverse	ACCTCCAGCCGCAGTGCC	CCGCGGTGCCCCTGCCT	AAGCGGAGACTCTGGTGC
APOE(R158)	Forward	GAAGCGCCTGGCAGTGTACC	CTGCGCAAGCTGCG	TCGGCGCCCTCGCG
	Reverse	GGTACACTGCCAGGCGCTTC	GGATGGCGCTGA	GCCTCGCCTCCCACC

Supplementary Table 4. PCR conditions for each target site analyzed by Sanger sequencing.

Target	Initial denature time and temperature	Denature time and temperature	Annealing time and temperature	Extension time and temperature	Final extension time and temperature
		40 cycles			
Site-1	98°C, 45 seconds	98°C, 10 seconds	54°C, 5 seconds	72°C , 20 seconds	72°C, 10 minutes
Site-2	98°C, 45 seconds	98 °C, 10 seconds	56°C, 5 seconds	72°C , 20 seconds	72°C, 10 minutes
Site-3	98°C, 45 seconds	98°C, 10 seconds	56°C, 5 seconds	72°C , 20 seconds	72°C, 10 minutes
APOE(R158)	98°C, 45 seconds	98°C, 10 seconds	62°C, 5 seconds	72°C , 20 seconds	72°C, 10 minutes

Supplementary Table 5. List of primers used in this study to amplify off-target sites. Abbreviations: BG-OT = Off-targets associated with sg(BG), Site1-OT = Off-targets associated with sg(Site-1), Site2-OT = Off-targets associated with sg(Site-2), Site3-OT = Off-targets associated with Sg(Site-3).

Primer	Forward Sequence (5'→3')	Reverse Sequence (5'→3')
BG-OT1	GATGCGCTTCCGGAAGACC	GCTTCTTGAGCTTCTCAGCG
BG-OT2	GGTAGCATGTTCAGGCACCAG	CATCCCTAGTACCGAATCCCATATAGC
BG-OT3	CATCCTCCCACCTAAGCCTTTCAA	TTGAGTTAATAGCATTATAACAATTTCCACA
BG-OT4	ACTCCTTACAACCGGAAGGCAAAC	TGGACGTGGTGAAGCCCGTGGTG
BG-OT5	TAGGTCTCTAGGGGGCCTCTG	AGGCTGCCCAACAGCCCCACT
Site1-OT1	TCCCCTGTTGACCTGGAGAA	CACTGTACTTGCCCTGACCA
Site1-OT2	TGAGATGTGGGCAGAAGGG	TTGGTGTTGACAGGGAGCAA
Site1-OT3	GTCCAAAGGCCCAAGAACCT	TGAGAGGGAACAGAAGGGCT
Site1-OT4	GCTCATCTTAATCTGCTCAGCC	TCCTAGCACTTTGGAAGGTCG
Site1-OT5	AAAGGAGCAGCTCTTCCTGG	GTCTGCACCATCTCCCACAA
Site2-OT1	GTGTGGAGAGTGAGTAAGCCA	ACGGTAGGATGATTTCAGGCA
Site2-OT2	TTTTTTGGTACTCGAGTGTTATTCAG	CACAAAGCAGTGTAGCTCAGG
Site3-OT1	GGCATGGCTTCTGAGACTCA	CCCCTTGCACTCCCTGTCTTT
Site3-OT2	GAAGAGGCTGCCCATGAGAG	TTTGGCAATGGAGGCATTGG
Site3-OT3	GGTCTGAGGCTCGAATCCTG	CTGTGGCCTCCATATCCCTG
Site3-OT4	TTTCCACCAGAACTCAGCCC	CCTCGGTTCCTCCACAACAC
Site3-OT5	GCAGGGGAGGGATAAAGCAG	CACGGGAAGGACAGGAGAAG

Supplementary Table 6. List of primers used in this study for NGS analysis.

Primer	Forward Sequence (5'→3')	Reverse Sequence (5'→3')
Site-1	ATGTGGGCTGCCTAGAAAGG	CCCAGCCAAACTTGTCAACC
APOE(R158)	GGACGAGACCATGAAGGAGTTGAAGGC	CCACCTGCTCCTTCACCTCGTCCAG

Supplementary Table 7. PCR conditions for each target site subjected to NGS analysis.

Target	Initial denature time and temperature	Denature time and temperature	Annealing time and temperature	Extension time and temperature	Final extension time and temperature
		40 cycles			
Site-1	98˚C, 45 seconds	98˚C, 10 seconds	54˚C, 5 seconds	72˚C, 20 seconds	72˚C, 10 minutes
APOE(R158)	98˚C, 45 seconds	98˚C, 10 seconds	62˚C, 5 seconds	72˚C, 20 seconds	72˚C, 10 minutes

Supplementary Table 8. Comparison of editing efficiency using RoT-based approaches at the same target loci in this manuscript, Komar et al., and Koblan et al.

	**Figure 3E Standage-Beier et al.**			**Figure 5C Komar et. al Sci Adv. 2017 Aug 30;3(8)**			**Figure 1C Koblan et. al Nat Biotechnol. 2018 Oct;36(9):843-846**		
	Reporter of Transfection			No Reporter			Reporter of Transfection		
	Unsorted	Reporter-	Reporter+	Unsorted	Reporter-	Reporter+	Unsorted	Reporter-	Reporter+
**Site-1 (HEK 3)**	21.3±2.9	3.3±2.8	40.7±7.0	~45	N/A	N/A	~38	N/A	~55
**Site-2 (HEK 2)**	36.6±3.8	13.3±5.9	49.7±5.1	~35	N/A	N/A	~20	N/A	~38
**Site-3 (HEK 4)**	24.0±6.6	7.6±5.0	45.3±1.5	~45	N/A	N/A	~25	N/A	~40
